# Common Variants of the Prostaglandin-Endoperoxide Synthase 2 Gene and Hepatocellular Carcinoma Susceptibility

**DOI:** 10.1097/MD.0000000000001116

**Published:** 2015-09-04

**Authors:** Hong-guang Li, Fang-feng Liu, Hua-qiang Zhu, Xu Zhou, Jun Lu, Hong Chang, Jin-hua Hu

**Affiliations:** From the Department of Hepatobiliary Surgery, Shandong Provincial Hospital affiliated to Shandong University, Jinan, Shandong, China (HGL, FFL, XZ, JL, HC); and Department of Gastroenterology Surgery, Shandong Provincial Hospital affiliated to Shandong University, Jinan, Shandong, 250021, China (JHH).

## Abstract

Hepatocellular carcinoma (HCC) is a heterogeneous disease with substantial genetic constitution. Previous work has evaluated the effect of prostaglandin-endoperoxide synthase 2 (*PTGS2*) variants (−765G/C, −1195A/G, and +8473T/C) on the development of HCC, but the conclusions are inconsistent. We conducted a meta-analysis in this work. Data from 7 case–control studies were combined to assess the association between *PTGS2* variants and HCC. The risk of HCC (OR and 95% CI) was estimated using either the fixed- or the random-effects model according to the Q test. No significant association was identified for −765G/C and +8473T/C. However, we identified a significantly decreased risk in relation to the GG genotype of −1195A/G (OR = 0.70, 95% CI = 0.50–0.98 for GG versus AA). We also observed a similar decrease (OR = 0.47, 95% CI = 0.23–0.95 for GG versus AA) in Caucasian samples. Variant −1195A/G in the promoter *PTGS2* may protect against the malignant progression of HCC. This significant association suggests that −1195A/G could be used as a biomarker of HCC.

## INTRODUCTION

Hepatitis virus infection, hepatitis B virus and hepatitis C virus in particular, is a known cause of hepatocellular carcinoma (HCC), an end-stage complication of fibrotic and chronic inflammatory liver disease.^1–4^ The interferon alpha therapy is widely used to treat hepatitis B virus- or hepatitis C virus-induced chronic viral hepatitis by changing the natural course of the diseases that will eventually advance to HCC.^5,6^ The interferon alpha treatment, however, is not effective for all patients. It works only among sustained responders by repressing replication of hepatitis virus.^7,8^ There is wide difference in individual susceptibility toward HCC among hepatitis virus-infected populations,^9,10^ suggesting the possible role of genetic variation in the disease pathogenesis.

Prostaglandin-endoperoxide synthase 2 (*PTGS2*) is a pro-inflammatory enzyme induced by prostaglandins involved in cell proliferation, tumorigenesis, progression, and metastasis.^11^ The inducible enzyme enables the transformation of arachidonic acid into prostaglandins. The *PTGS2* gene, also known as cyclooxygenase-2 (*COX-2*), is situated at human chromosome 1q25.2-q25.3. It is suggested that increased serum PTGS2 levels lead to upregulation of the prostaglandin EP1 receptor, which in turn decreases PTGS2 levels through certain pathways.^12^ The levels significantly increase in cancerous tissues and this increase may constitute a mechanism that facilitates carcinogenesis.^13^ In addition, cyclooxygenase inhibitors could downregulate serum concentrations of potent angiogenic factors, thus suppressing angiogenesis and precluding tumor growth.^14^ Therefore, the *PTGS2* gene may contribute to the progression of human cancer.

A case–control study of Chinese samples suggested that *PTGS2* −765G/C and −1195A/G increase the genetic susceptibility to HCC, with −765GC and −1195AA associated with 2.89 and 1.57 times higher risk, respectively.^15^ Conversely, a decreased risk was detected for −765G/C and no association was found for −1195A/G and +8473T/C in Turkish samples.^16^ The controversial results are probably caused by sampling variance and population heterogeneity. In this study, meta-analysis was used to assess the association of the variants in the promoter region of the *PTGS2* gene with HCC susceptibility.

## METHODS

### Literature Search Strategy, Inclusion and Exclusion Criteria, and Data Extraction

This study was approved by the Institutional Review Board of Shandong Provincial Hospital affiliated to Shandong University, Jinan, Shandong, China. Literature searches were performed through a 3-stage strategy to identify all potentially relevant papers. At stage 1, we systematically searched Embase, PubMed, Science Direct, and Wangfang databases, with the last search completed on November 16th, 2014. The key words including PTGS2, cyclooxygenase-2, HCC, liver cancer, and polymorphism were used. Two reviewers scanned the retrieved articles by title, abstract, and full texts whenever necessary to single out all case–control studies examining the association between one or more *PTGS2* variants and HCC incidence. At stage 2, we checked the reference lists of each case–control study to gain new data. At stage 3, we contacted the corresponding author of an Italian study by e-mail, as the genetic data were incompletely reported in the original article.^17^

The inclusion criteria designed for the human case–control studies included: used at least one of the *PTGS2* variants to estimate the risk of HCC, clearly reported the genotype frequency, and the samples used in the study must be unique without any subsequent updates; if there were, the study with the largest sample size was considered in the final analysis. We excluded the studies for various reasons: only HCC patients were investigated, systematic reviews, animal studies, editorials, summary abstracts without complete data, and case reports.

Data on the following items were recorded for the case–control studies: surname of first author, journal and year of publication, study design, country of origin, ethnicity, proportion of men and women, selection of controls, methods used to determine the genotype of *PTGS2* variants, genotyped cases and controls, and count of genotypes. Data were collected by 2 independent reviewers and subsequently checked by a third reviewer. In case of discrepancies, an expert in this filed was invited to make a final decision.

### Quality Assessment

We evaluated Hardy-Weinberg equilibrium (HWE) in control populations using the X^2^ test to assess the quality of the studies included in this meta-analysis.^18^ A *P* value lower than 0.05 indicated significant HWE deviation. The studies were categorized into the high-quality group when *P* > 0.05; otherwise, they were considered low-quality studies.

### Statistical Analysis

The fixed-effect model proposed by Mantel and Haensze^19^ and the random-effect model described by DerSimonian and Laird^20^ were properly used to estimate the risk of HCC (OR and 95% CI: odds ratio and 95% confidence interval). The former model was performed when the Chi-square based Q statistic test and I^2^ statistic indicated no notable heterogeneity between studies (*P*_Q-test_ > 0.05 and I^2^ < 50%);^21^ otherwise, the latter model was used. Subgroup analyses were performed according to ethnicity for *PTGS2* −1195A/G. Sensitivity analyses were used to check if the single studies had significant influence on the combined risk estimates. Publication bias was determined using the funnel plots and the Egger liner regression test.^22,23^ All 2-sided *P* values less than 0.05 were considered significant. Statistical analyses were done using the Stata software package (version 12.0; Stata Corporation, College 162 Station, TX) and the meta package for R (version 3.0.3; the R Foundation for Statistical Computing, Tsukuba, Japan).

## RESULTS

### Characteristics of Studies

**FIGURE 1:**
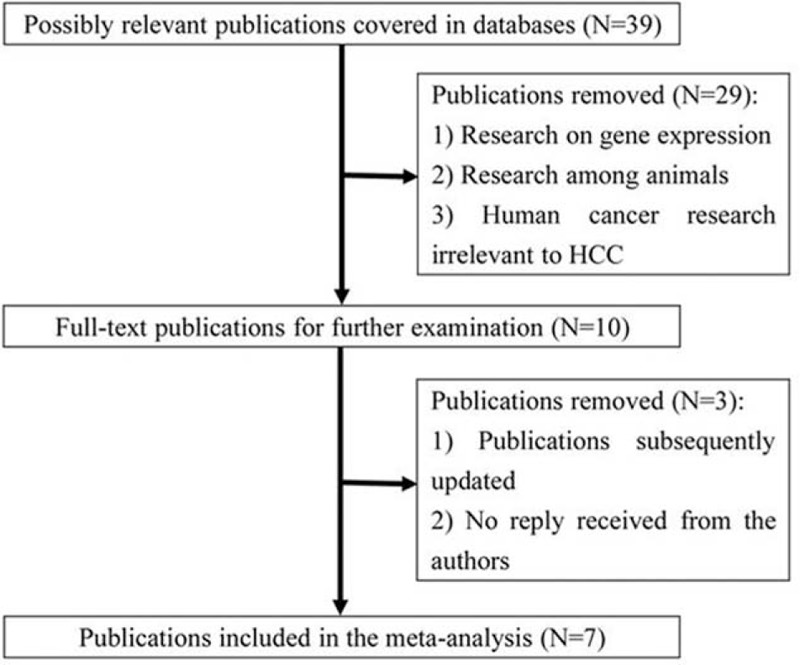
Flow chart for primary selection in this meta-analysis.

A total of 39 records were initially identified, as shown in Figure 1. We scanned all titles and abstracts, excluding 29 articles as a result of expression-based study, using an animal model to investigate the etiology of HCC or irrelevant human cancer research. We further examined eligibility of the remaining 10 full texts. Three studies were excluded, because 2 were subsequently updated,^24,25^ 1 did not provide sufficient genetic data and no reply was received after contacting the authors.^17^ The pooling dataset therefore comprised 7 studies.^15,16,26–30^ Of these, 5 studies analyzed −765G/C, 6 investigated −1195A/G, and 3 studied +8473T/C. For −765G/C, 2 studies were conducted in Caucasians and 3 in Asians, with Akkiz et al deviating from HWE. With respect to −1195A/G, there were 4 Asian studies and 2 Caucasian studies, and no HWE deviation was detected. In terms of +8473T/C, 2 studies employed Asians and 1 Caucasians. Two types of methods were used in determining the genotypes of *PTGS2* variants, including PCR-RFLP (polymerase chain reaction-restriction fragment length polymorphism) and PCR-TaqMan. Genotype frequencies between cases and controls and quality status are presented in Table 1.

**TABLE 1:**
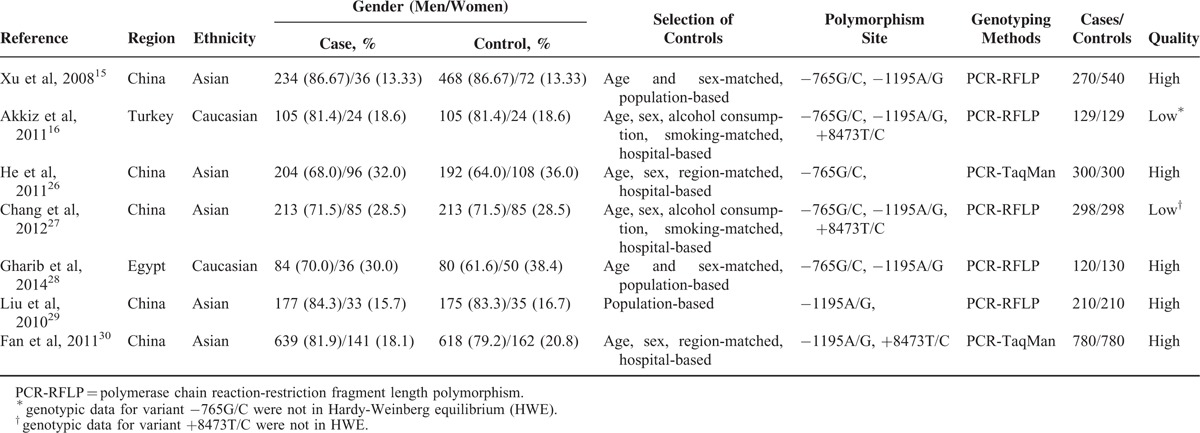
Main Characteristics Collected for Eligible Studies

### Association Between −765G/C and HCC Susceptibility

**FIGURE 2:**
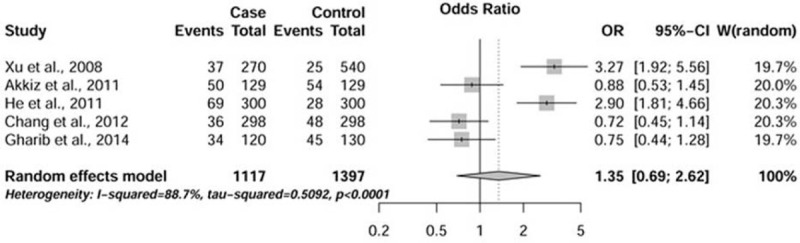
Forest plot of HCC susceptibility associated with variant −765G/C (CC + GC versus GG). The squares and horizontal lines correspond to the study-specific odds ratio (OR) and 95% confidence interval (CI). The area of the squares reflects the weight (inverse of the variance). The diamond represents the summary OR and 95% CI. HCC = hepatocellular carcinoma.

As shown in Table 2, there was no relationship observed between the presence of variant −765G/C and genetic susceptibility of HCC. The forest plot created for CC + GC versus GG model is shown in Figure 2. Likewise, no significant effect was revealed in the analysis excluding the low-quality study (data not shown). Large between-study heterogeneity was indicated in all analyses. Subgroup analysis suggested that ethnicity was the major source of the nonhomogeneous results (data not shown).

**TABLE 2:**
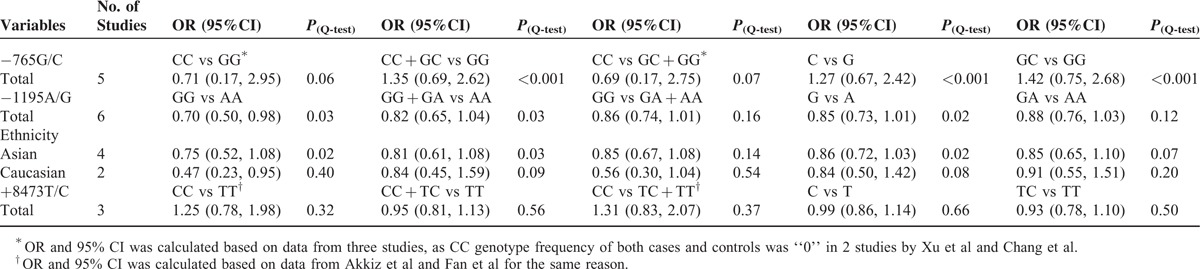
Main Results of the Meta-Analysis

### Association Between −1195A/G and HCC Susceptibility

**FIGURE 3:**
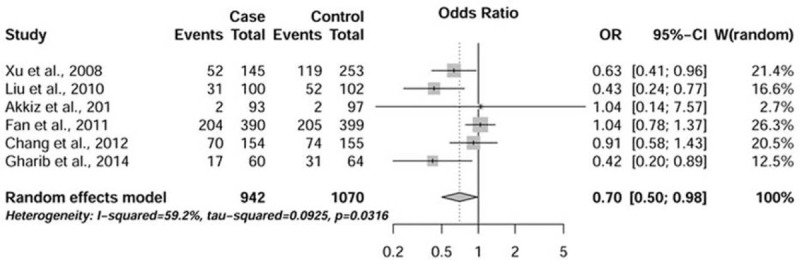
Forest plot of HCC susceptibility associated with variant −1195A/G (GG versus AA). The squares and horizontal lines correspond to the study-specific odds ratio (OR) and 95% confidence interval (CI). The area of the squares reflects the weight (inverse of the variance). The diamond represents the summary OR and 95% CI. HCC = hepatocellular carcinoma.

Meta-analysis of −1195A/G and HCC susceptibility was performed among 1807 patients and 2089 control subjects. We found 30% decreased susceptibility of HCC in relation to the GG genotype using the GG versus AA model (OR = 0.70, 95% CI = 0.50–0.98, Figure 3). Further analysis among Caucasian also showed a markedly decreased risk (OR = 0.47, 95% CI = 0.23–0.95 for GG versus AA, Table 2). Subgroup analyses showed heterogeneous results in Asians and homogeneous results in Caucasians, suggesting that the significant heterogeneity may primarily stem from the Asian studies. We then performed sensitivity analyses and found low heterogeneity when excluding the study by Fan et al.^30^ In addition, analysis restrained to high-quality studies showed no significant association in Asian (data not shown).

### Association Between +8473T/C and HCC Susceptibility

Compared to the TT genotype, the CC and TC combined were not more likely to develop HCC (OR = 0.95, 95% CI = 0.81–1.13). The ORs calculated using other genetic models indicated the same results. No analyses showed evidence of significant heterogeneity (Table 2).

### Bias Diagnostics

**FIGURE 4:**
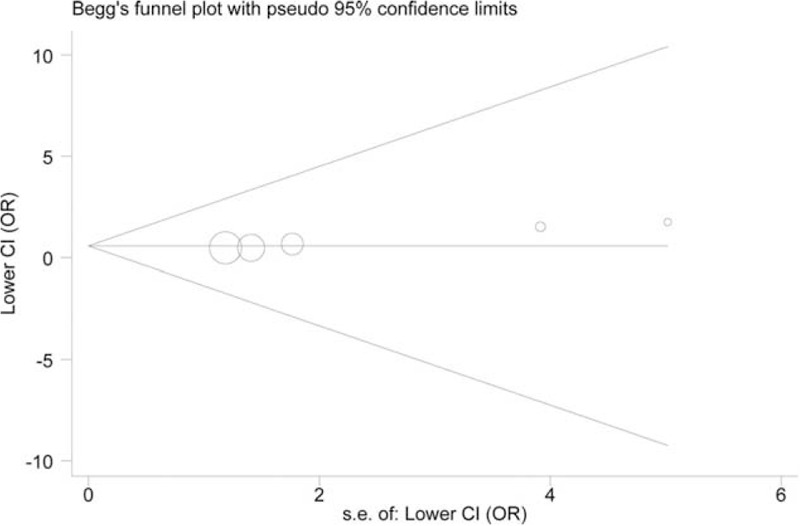
Begg plot for the assessment of potential publication bias for variant −765G/C.

**FIGURE 5:**
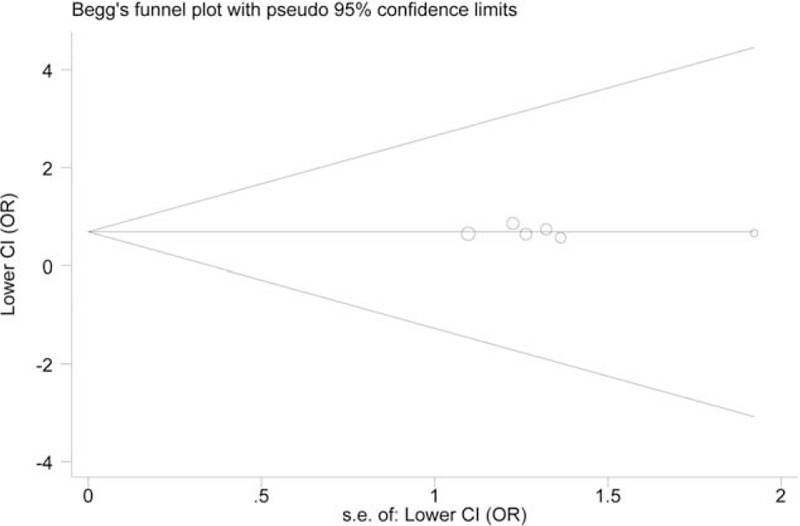
Begg plot for the assessment of potential publication bias for variant −1195A/G.

We assessed the publication bias for −765G/C and −1195A/G. Symmetrical distribution was observed in all funnel plots (*P* > 0.05). However, the Egger test demonstrated evidence of high possibility of publication bias in the GC versus GG model of −765G/C (*P* = 0.001, Figure 4). Figure 5 shows the funnel plot constructed for −1195A/G using the GG versus AA model (*P* = 0.811).

## DISCUSSION

This study, for the first time to our knowledge, has examined the association between *PTGS2* gene variants (−765G/C, −1195A/G, and +8473T/C) and HCC susceptibility by the use of meta-analysis. A total of 7 case–control studies were incorporated. Of the *PTGS2* variants studied, only one, corresponding to −1195A/G, was found to be associated with HCC. We noted that the presence of a GG genotype may reduce the risk to develop HCC, especially in Caucasians. Although the statistical power is relatively stronger compared to the previously published studies, some findings should be interpreted with caution due to the large heterogeneity and significant publication bias.

Several groups have investigated the hypothesis of a relationship between the variants in the *PTGS2* gene and HCC. The first case–control study was published by Xu et al in 2008.^15^ In this analysis, 270 HCC patients and 540 controls subjects of Chinese race were analyzed to evaluate the effects conferred by −765G/C and −1195A/G, which were reported to be associated with significantly increased risk of HCC (OR = 1.57 and 2.89, respectively). In the independent studies following Xu et al, some replicated the significant association, while others did not. For example, Liu and Lin^29^ investigated the variant −1195A/G only and found 1.38-fold increased risk among 420 Chinese samples (210 cases and 210 controls) with the −1195A allele. In contrast, Fan et al^30^ showed no evidence supporting that −1195A/G is a risk factor for HCC, even though they identified 50% decreased risk associated with +8473T/C among 780 cases and 780 controls of Chinese ancestry. Such controversial results were also indicated in Caucasian samples.^16,28^ The wide disparity in the reported results may result from certain aspects, including methodological errors (eg, selection bias, genotyping errors, and population stratification), different ethnic groups, and limited sample size. Other plausible factors, such as poor study design, may also contribute to the inconsistency.

In our study, we demonstrated that the *PTGS2* promoter variants being investigated, with the possible exception of −1195A/G, were not associated with the risk of developing HCC. Our findings were in agreement with most published papers, including a recent meta-analysis by Bu et al.^31^ This meta-analysis evaluated the effects of −1195A/G only and identified a statistically significant increase in the risk of HCC (OR ranged from 1.26 to 1.45). We identified a new Caucasian study and found significant effects in these populations. In addition, we assessed the association for −765G/C and +8473T/C, showing no evidence of a major effect.

The results of the present analysis seem to contradict the previous experimental studies. Akkiz et al^16^ reported that −765CC genotype downregulates the expression of *PTGS2*; it is the decreased promoter activity that may represent an important mechanism to explain the significant reduction in the risk of HCC. It is suggested that aberrant expression of the *PTGS2* gene induced by the genetic variations in its promoter region would result in chronic immune activation and inflammation, uncontrolled cell growth, suppressed apoptosis, precancerous lesions, and subsequent tumor formation.^32,33^ Therefore, the *PTGS2* genetic variants are likely to modulate HCC predisposition through up- or downregulating the expression of the gene they are mapped on.

There are several strengths in the meta-analysis. The first strength refers to the comprehensive evaluation of the association between *PTGS2* variants and HCC susceptibility. We included all available data for the commonly studied variants to provide compelling evidence for the associations that remain elusive. The second strength is that we identified no genetic contributions for −765G/C and +8473T/C, which was not reported in the early meta-analysis. Nevertheless, there was some evidence of notable between-study heterogeneity, and publication bias possibly caused by the failure to include pertinent unpublished reports. Some of our findings may be affected. This is the first limitation that should be taken into consideration when interpreting the present results. Second, the sample size is too low for some variant sites and subgroups, leading to less definite conclusions for some findings. Third, as none of the included studies stated adjusted ORs, the genetic association of interest was evaluated using unadjusted ORs. Finally, both environmental and inherited genetic risk factors are involved in the etiology of HCC. Nonetheless, the effect of gene–environment interactions was not assessed and this may finally influence the findings we demonstrated in the present study. These limitations emphasize the need for continued clinical and basic research to provide new evidence for the molecular mechanisms underlying HCC incidence.

In summary, our meta-analysis suggests that the development of HCC may be associated with *PTGS2* variant −1195A/G, but not −765G/C or +8473T/C. A larger study which takes gene–environment interactions and role of confounding factors into account is expected to validate our findings and to determine the role of these variants in HCC pathogenesis.
